# 

**Published:** 1893-02-18

**Authors:** 


					CONTENTS.
5\5r5?lleSjre to the Press
323
Ohtaie
^edlcS8i? India.
?The Bengal Presidency
? 324
h.V,ulas in 10 xne -fress ?
^?al Histnv^" v 1-~Tlla Bengal Presidency
????otow m7a *??** tJie Earliest Times.
By E. T.
"rtBisnSi, ,ry from the
cJ?** M.B.Oxon.
XLIII.?The Medical Profession
E?C?l^dl0 K' M;B-?fon.
&?hS5??4li A?a' . on\ XljUi-7T
d ??SBiSy?wll?ory an<* Research
326
4e aoswtar,y ^eory
St. TjjnC . Clinic-
?Empyema
331
^AlOPaTL,?ap"AL'-Empvema . .
D??njunoti?j^ Hospital, Mooefields.?
?The Treatment of
SSb&SSi
332
^0?Ar H
pPever t_Heen Hospital, Liveepool.
-Treatment of Typhoid
Fevej
S3S
Vfor .  ijivLuru
t " "
^UconB pSM.AIiT> Edinburgh.?
Operative Treatment of
Mucous p*, .aet> Edinb
UUB Polyp, of the Nose
334
The Parson and the Public House
- 327
Everybody's Fag's
Dinmv:nM
328
Annotations.
-Invertebrate Medicine?Plumbing and Cholera?
A Word for Poor Physio
329
Notes and news
330
The institutional Workshop?
Within tub Hospitals.?
Huddersfield Infirmary?
The Durham
Oouuty Hospital
S35
Hospital Oonstbucxion.?
Lying-in Hospital at Helsingfors.
Finland
? 335
The Story of the Insane from Year to Year
Hospital opinion.?
Hospital Unoharity
637
Scraps and Gleaning-s - 3S8
The Student of Medicine.?Vacancies?Notes from the Schools
?af EXTRA SUPPLEMENT.?THE NURSING MIRROR.
World-The Empress Frederick
l0Wss"\r Assurance?Workers Wanted?Comforts in
atilty nftirvfn*!lDK at Tnnbridjje Wells?Mind and Body?
Sydney?B)1???1?*,~Pro^ress at Londonderry?Nursing at
,?naowed Itoms-A. Disabled Nurse-" The Hospital
*a*aSd Bed r "tiou ?u?r .XUD r""*:""*.
Oowumfntinn11STlmPti0n- n--SymPt0mS. ***
^?iatmPe^tments "? ? - ? ? - ' '
^ - ts?Qur Examinations
cslix
Croupous Pneumonia oliii
" Vlctoriahaus fur Krankenpflege" .... oliii
Everybody's Opinion.?Useful Hints Wanted?The Best
Nursei?England versus America?The Late Mr, Hewer? ,
Workhouse Infirmaries?" The Hospital " Endowed Bed ? cliv
The History of Nursing1 - olv
Por Beading" to the Sick.?The Star civ
Wants and Workers civ
Some Australian Experiences clvi
Notes and Queries - -  clvi
oli
cli
clii

				

